# RMS: a platform for managing cross-disciplinary and multi-institutional research project collaboration

**DOI:** 10.1186/s12911-014-0106-6

**Published:** 2014-11-30

**Authors:** Jake Luo, Carolyn Apperson-Hansen, Clara M Pelfrey, Guo-Qiang Zhang

**Affiliations:** Center of Biomedical Data and Language Processing, University of Wisconsin Milwaukee, 2025 E Newport Avenue, Northwestern Quadrant-B, Room 6469, Milwaukee, Wisconsin 53211 USA; Center for Clinical Investigation, BRB 109, Case Western Reserve University, 10900 Euclid Avenue, Cleveland, Ohio 44106 USA; Division of Medical Informatics, 2103 Cornell Road, Wolstein Research Building, Room 6128, Ohio, 44106 USA; School of Medicine, Case Western Reserve University, Cleveland, Ohio USA

**Keywords:** Biomedical research, Organization & administration, Research collaboration, System design and development, Collaborative research, Communication networks, Systems integration, Data-driven analysis

## Abstract

**Background:**

Cross-institutional cross-disciplinary collaboration has become a trend as researchers move toward building more productive and innovative teams for scientific research. Research collaboration is significantly changing the organizational structure and strategies used in the clinical and translational science domain. However, due to the obstacles of diverse administrative structures, differences in area of expertise, and communication barriers, establishing and managing a cross-institutional research project is still a challenging task. We address these challenges by creating an integrated informatics platform to reduce the barriers to biomedical research collaboration.

**Results:**

The Request Management System (RMS) is an informatics infrastructure designed to transform a patchwork of expertise and resources into an integrated support network. The RMS facilitates investigators’ initiation of new collaborative projects and supports the management of the collaboration process. In RMS, experts and their knowledge areas are categorized and managed structurally to provide consistent service. A role-based collaborative workflow is tightly integrated with domain experts and services to streamline and monitor the life-cycle of a research project. The RMS has so far tracked over 1,500 investigators with over 4,800 tasks. The research network based on the data collected in RMS illustrated that the investigators’ collaborative projects increased close to 3 times from 2009 to 2012. Our experience with RMS indicates that the platform reduces barriers for cross-institutional collaboration of biomedical research projects.

**Conclusion:**

Building a new generation of infrastructure to enhance cross-disciplinary and multi-institutional collaboration has become an important yet challenging task. In this paper, we share the experience of developing and utilizing a collaborative project management system. The results of this study demonstrate that a web-based integrated informatics platform can facilitate and increase research interactions among investigators.

## Background

A recent study on 19.9 million scientific publications shows that team science is dominating knowledge production compared to solo authors [1]. Hall et al. compared trans-disciplinary center grants with investigator-initiated grants and discovered that trans-disciplinary grants have a higher publication rate and more coauthors [2]. Cross-disciplinary collaboration is one of the key components of team science [3–5]. A longitudinal study revealed that research collaboration not only effectively promoted more research output, but also significantly correlated to the quality of papers [6]. Another study also discovered that lack of cross-disciplinary collaboration was a significant barrier to clinical outcome research [7]. Several emerging areas in the biomedical science domain, including Clinical Research Informatics [8,9] and Medicine 2.0 [10], have identified collaboration as one of the key driving factors of successful research.

Having realized the importance of research collaboration, research agencies have gradually begun supporting more and more projects that are capable of bringing together experts from multiple disciplines and of exerting broader impact to multiple domains [11,12]. The National Institutes of Health (NIH) Roadmap [13] established new aims to improve Biomedical Research, in which the Clinical and Translational Science Awards (CTSA) [14] program is the most important feature. One strategic goal [15] for creating the CTSA program was to bridge the gaps between different disciplines and remove barriers to communication, which can increase the efficiency of collaboration between the basic science researcher, clinical scientist and practicing physician. Cleveland’s CTSA, the Clinical and Translational Science Collaborative (CTSC), was an early recipient of the NIH funding for the CTSA award. The consortium is actively involved in building infrastructure to support clinical and translational research at the Case Western School of Medicine, the Cleveland Clinic, University Hospitals Case Medical Center, MetroHealth Systems, and the Louis Stokes VA Medical Center and has had a positive impact on many aspects of the affiliated institutions [16]. The CTSC designates Research Concierge Services (RCS) as the entry point for accessing the resources and expertise within the CTSC as well as externally. The goal of the RCS is to assist investigators navigating the cross-disciplinary research process and to provide consultation support at each phase of clinical and translational research. However, like many multi-institutional organizations, the CTSC has a complex five-partner structure and so faces challenges with respect to coordinating the experts and resources located at different sites for collaborative research.

The RCS discussed the challenges with experts of the CTSC Biomedical Research Information Management (BRIM) Core which has a special focus on providing informatics infrastructure and developing computerized tools and systems to support the multi-faceted clinical and translational research. The BRIM and the RCS found that there was a strong need for an informatics platform that can systematically tackle the management challenges of collaborative projects. Four major requirements were identified as desirable for reducing the barriers to collaborative research projects.

The first requirement is integrating institutional resources and expertise to support research project development and enable collaboration. For example, the BERD core (Biostatistics, Epidemiology, and Research Design Core), comprising experts in epidemiology and biostatistics across partner institutions, provides support for the development of research protocols and statistical analysis plans. It is not always easy to access these resources due to the diverse expertise, constant changes, disparate locations and various administration structures. Furthermore, many investigators may not be aware of the range of services provided by the CTSC cores. Hence there is a strong need to aggregate the services in an informatics system so that investigators can search and acquire collaborative expertise easily.

The second requirement is to streamline the process of obtaining expert support and technological consultations. When investigators are conducting new research projects, they often need help from other experts; for example, they may need to consult the Bioethics Offices about on ethical regulations and before designing a statistics plan with the BERD core. Coordinating the efforts of different experts and following up on the progress of several threads is time-consuming and often requires sophisticated project management skills. Our goal is to reduce such barriers by implementing a streamlined workflow to allow the creation, monitoring, and discussion of collaborative projects even when the collaborators come from different cores or are located at different CTSC sites.

Third, as the “front door” to accessing the resources and expertise, the RCS requires an informatics system that can manage the roles of CTSC members and monitor the status of research projects. The RCS is responsible for many administrative tasks, such as helping investigators initialize collaboration with experts, describing the projects to collaborators, assigning participant roles, and tracking the status of ongoing projects. This infrastructure required a system designed with sophisticated project administration functionalities, such as authorizing experts according to their expertise and responsibilities and monitoring the progresses of projects.

Last, the ability to analyze project performance and report core activities located at many different sites to the CTSC leaders is a challenging task. Senior leaders of the institutions need evidence-based evaluation based on accurately documented project data. It is important to maintain a research project repository that can be used to summarize the activities, analyze resource usage, and predict trends. These types of information would greatly improve the evaluation process and provide a more global view of research activities for the CTSC leadership in important decision making, such as resource allocation.

**Figure 1:**
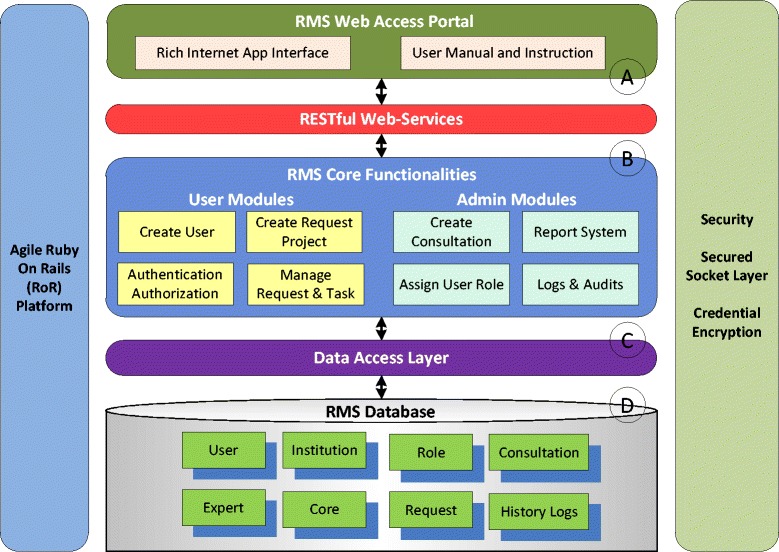
**System architecture of RMS: (A) web portal, (B) core functionalities, (C) data access layer, and (D) RMS database.**

Stokols et al. [4,5] pointed out that informatics infrastructure is an important factor for research collaboration. Team Science Toolkit [17,18] is a collaboration tool that supports the practice and studies of team science. The Team Science Toolkit project is supported by the National Center Institute. Researchers can share and post team science resources on the website. For example, SciVal Experts [19] and VIVO [20] are listed as infrastructures to index domain expert information. The website also features an expert directory. However, currently there is still the lack of infrastructure to support the initialization and management of collaborative projects. In this paper, we developed an integrated infrastructure called the Request Management System (RMS) to address the needs of managing collaborative biomedical research projects (See Figure 1). RMS was implemented based on a highly modified derivative of the Multi-Modality & Multi-Resource Informatics (MIMI) framework [21,22]. MIMI is a web-based framework that supports customizable role management and resource allocation.

## Implementation

### System architecture

The system infrastructure of the RMS platform is illustrated in Figure 1. We chose open-source technologies to support platform development and Ruby on Rails [23] was chosen as the programming language for the project.

Users access all the RMS functionalities through a one-stop web portal (See Figure 1-A). The portal provides an interactive interface [21,22] using Ajax [24] technology for both project management and system administration. The *rich internet application* (RIA) techniques are capable of providing services through the web browser without the user having to download and install software packages. To support new investigators who are not familiar with the system, two instruction manuals (see Notes section) are provided at the portal entrance. Two user manuals are provided: 1) a User Instruction Manual to describe the functions of RMS, and 2) a Quick Start Guide to illustrate the process of submitting the first request to registered domain experts.

Through a layer of RESTful web services [25], the interface calls the RMS core functionalities to support user requests (See Figure 1-B). The RMS business logic can be classified into two categories based on the role of users. The *User Module* handles tasks sent by an investigator who is seeking to initialize or monitor a project. The *Admin Module* provides convenient functions for administrators to manage the RMS system. The modules are linked as a collaborative workflow to support streamlined user operations. Another important responsibility of the business logic layer is to keep track of the business entities. The business entities are stored in a database through the data access layers (See Figure 1-C).

The RMS database can store thirty-five business entities. In Figure 1-D, we show the eight most important entities. The *User* entity represents an RMS user who has been assigned user properties, including affiliation, contacts, account credentials, and most importantly, a role. The role restricts the privileges of a user and defines authorized actions on RMS, such as viewing, editing, and deleting information. The *Institution* entity represents institutional partners. A *Core* includes a group of experts who share similar research interests and devote their time to serve the community with professional skills. A *Project* is created by an investigator to seek consultation and establish collaboration with core experts. If a project is accepted, the investigator can create multiple *Tasks* with different core services and keep track of task progress. For administrators, the *Report* entity and the *History Log* entity help provide an overview of the system conditions. All the business entities are individually customizable and adaptive to change. Hence, we can deploy the system to a new institution by adding organizational structure and defining local services using the system management tools.

The security of the RMS system is managed on three layers. On the system level, it is protected by a firewall that prevents access from unauthorized parties. On the network communication level, the connections between users and server are encrypted by a Secured Socket Layer (SSL) that prevents man-in-the middle attacks. For user credentials, user passwords are also hashed by a 256 bit Secure Hash Algorithm (SHA) to encrypt password. To maintain service integrity and content provenance, a history log is also used to keep track of user activities.

### Collaborative workflow

Several studies have identified a lack of tools to manage and keep track of project targets and trajectories is the primary technology barrier impeding research collaboration [26,27]. RMS provides a solution to managing research collaboration by using a workflow engine. The workflow offers investigators an integrated informatics tool to manage the processes, contents, discussion and administration across institutions.

**Figure 2:**
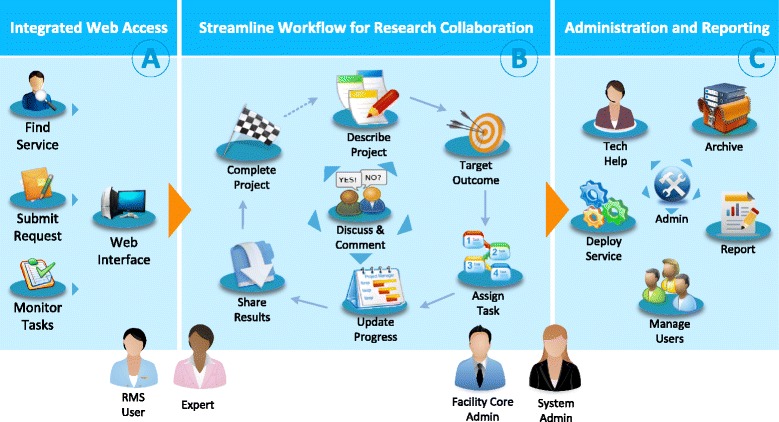
**Supporting collaborative biomedical research using RMS: (A) integrated web portal, (B) streamline workflow, and (C) administrative and reporting.**

The RMS workflow is shown in Figure 2. After logon to the portal, an investigator can browse through the Core service hierarchies to find services that satisfy the research need. Once the investigator identifies useful services or experts, they can submit an initial request by filling out an online form to describe the project, including the project title, summary and grant. The investigator also needs to clearly specify the expected study outcomes (See Figure 2-B). The top five requested outcomes are: *Advice and Information*, *Abstract or Poster*, *Peer Manuscript Review*, *Grant Submission Support*, and *Survey Development*. A new task is created to assign the collaboration request to an appropriate expert. The requester can discuss the project with the expert and set up task deadlines, and the expert can manage the updating of the completion times and work hours for the task. To support a large project that consists of a team of experts, the investigator may send multiple tasks to different experts to establish multi-faceted collaboration. Results and files are then shared among collaborators. The investigators and experts are also encouraged to discuss task progress on the RMS platform during the life-cycle of the research project.

On the system administration side (See Figure 2-C), the RMS platform integrates comprehensive tools to support the daily administration tasks. Core administration can create, change, and archive their services and assign domain experts to support consultation services. RMS also implements tools to support the daily operation of the RCS in a way that maximizes operational efficiency. For example, the user management tool provides methods for assigning roles, updating information, deactivating a user, or designating a person as a proxy. Another important tool is the report system, which sends reports to all the business entities, including reports related to overdue tasks, total requests, and active services. We developed an advanced Ad-hoc report system that allows administrators to organize information on any combination of business entities to generate a report. RMS has been keeping track of a large amount of CTSC resources and activities; hence it also serves as an analysis tool for CTSC leaders to enhance decision making. The reports generated by RMS have been used to support the evaluation of the program in an objective and measurable way.

## Results

### RMS members

Table 1 shows that up to June-01-2013, RMS had accumulated 1,520 users. Most users are members of CTSC partner institutions, including Case Western Reserve University (516), University Hospitals Case Medical Center (383), Cleveland Clinic (228), MetroHealth (230) and Louis Stokes VA Medical Center (22). There are also 31 users who come from other non-CTSC entities, such as the American Diabetes Association, the University of Chicago, and Weill Cornell Medical College. In total, users are from 36 different institutions. This result shows that RMS has already attracted a considerably large group of users from different external to the CTSC. The main goal of RMS is to facilitate investigators to establish collaborative projects and manage the collaboration process. For the CTSC, the target is to create more collaborative projects. In Result section 3, we provided evidence to show the growth of projects in RMS.

**Table 1:** **RMS users’ institutional affiliation**

**Institution**	**Number of RMS members**
Case Western Reserve University	516
University Hospitals/Case Medical Center	383
Cleveland Clinic	338
Metro Health Medical Center	230
Louis Strokes VA Medical Center	22
Others	31
**Total Institutions: 36**	**Total Members: 1520**

### RMS service summaries

Table 2 summarizes the expert services provided in each of the CTSC Cores. Thus far, the twelve cores have provided 176 different types of collaborative services on the RMS platform. Due to the diverse research interests and specialties in the CTSC, experts in different cores provide different kinds of services. Some cores, such as the *Biostatistics, Epidemiology, and Research Design* (BERD) core, and the *Biomedical Research Informatics Management* (BRIM) core, naturally have more collaboration due to the high demand for their expertise. Cores maintaining major CTSC resources have also received a lot of requests, such as the *Pilot Grant Program* and the *Practice-Based Research Network Shared Resource*. Over 4,800 tasks have been finished by 380 experts. In RMS, users can contribute their expertise to the community using their “expert” role; they can also seek help from other users and act as a “requester.” A user could have multiple roles at the same time depending on the projects they participate in and their system privileges. This creates a dynamic collaboration environment. Among all the registered 1,520 users, 380 (25%) of them have contributed their domain knowledge and time to serve other users. The results indicate that RMS has integrated core expertise and resources to effectively support a diverse range of collaborations.

**Table 2:** **Summary of RMS services**

**Research core/facility**	**Service type**	**Task**	**Expert**	**Admin**
Administration and Governance	6	295	23	7
Behavioral Science Measurement Resource	2	28	9	4
Bioethics and Regulatory Knowledge	27	74	31	5
Biomedical Research Informatics Management	13	841	30	11
Biostatistics, Epidemiology, and Research Design	20	1053	64	14
Clinical Research Unit Services	22	555	45	14
Community Research Partnership	8	148	17	5
Pilot Grant Program	4	781	34	4
Population Health and Outcomes Research	1	8	4	2
Practice-Based Research Network Shared Resource	30	664	23	5
Program Evaluation and Reporting	1	12	9	2
Translational Technologies Resources	42	359	91	26
**Total**	**176**	**4818**	**380**	**99**

### Growth of users and projects

**Figure 3:**
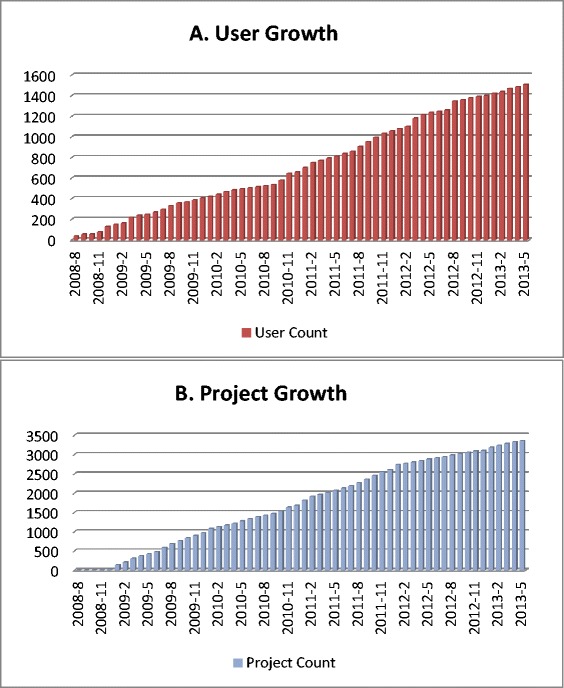
**Cumulative growth in number of RMS users (A) and cumulative growth in number of projects using RMS (B).**

Figure 3-A shows the cumulative growth of the RMS users since the launch of the system (from August, 2008 to May 2013). Similarly, Figure 3-B shows the projects submitted to RMS since launch. We can see a steady and consistent increase in both number of users and projects.

### Growth of research collaboration

**Figure 4:**
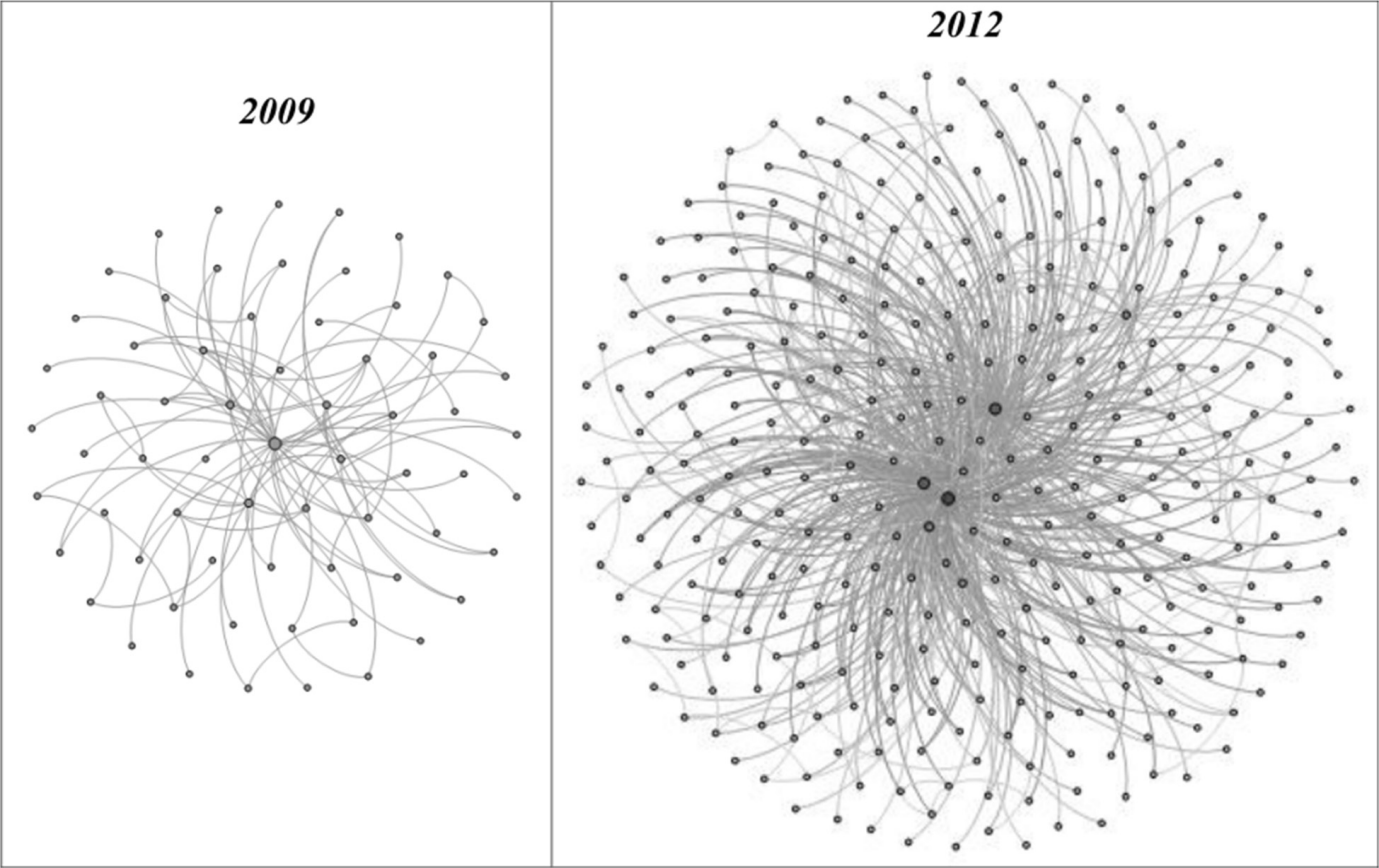
**Growth in number of research collaborations.**

Figure 4 shows the research collaboration network of the RMS users rendered by Gephi [28]. If two nodes have a connection, it means the two users have collaborated in at least one project. Nodes located close to the center of the diagram generally have more collaboration than the nodes at the edge of the diagram. The total count of the connections in 2012 (632 edges) was four times that of 2009 (122 edges), which demonstrates a significant increase in collaborations on RMS. The average connectivity degree of each RMS user also rose from 1.34 to 3.82, an increase of close to three hundred percent. These results indicate that RMS facilitated the increase of collaborations among members.

The total connection (collaborative work) increased 400% from 2009 to 2012. The results show a significant increase of collaborative work. As shown in Table 2, the collaboration service can be categorized in 176 different types within 12 research cores, and the collaborative task within each category is summarized. Future efforts will focus on evaluating the quality of collaboration facilitated through RMS.

### Cross-disciplinary collaboration

**Figure 5:**
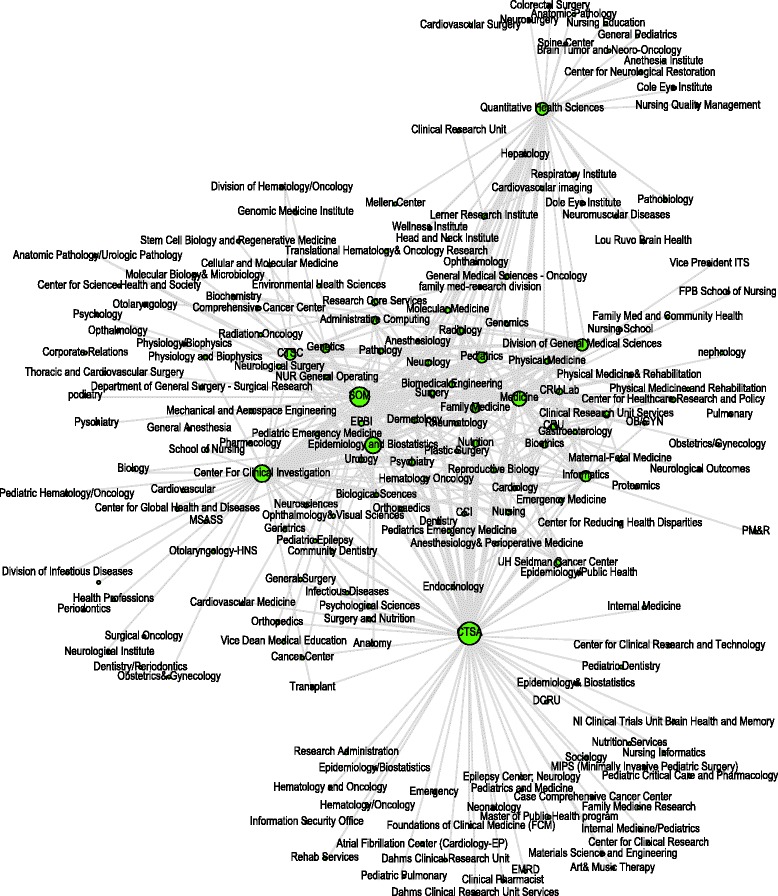
**Cross-disciplinary collaboration network.**

Figure 5 shows the cross-disciplinary collaboration network among RMS users. A node represents a research division of five of the CTSC partner institutes. Investigators belonging to the same division are *grouped into one node*. The size of a node represents the degree of connection between research divisions. The larger the node size, the more total collaboration the division has. An edge connecting two nodes represents collaborative projects between the two research divisions. Thicker edges mean more collaboration between the two divisions. The diagram shows 201 divisions and 606 collaborative connections. The average connectivity degree of each division is 5.98. This network and Table 2 shows that RMS has been supporting 176 different types of collaboration across 201 research divisions.

### Word cloud of project discussion notes

**Figure 6:**
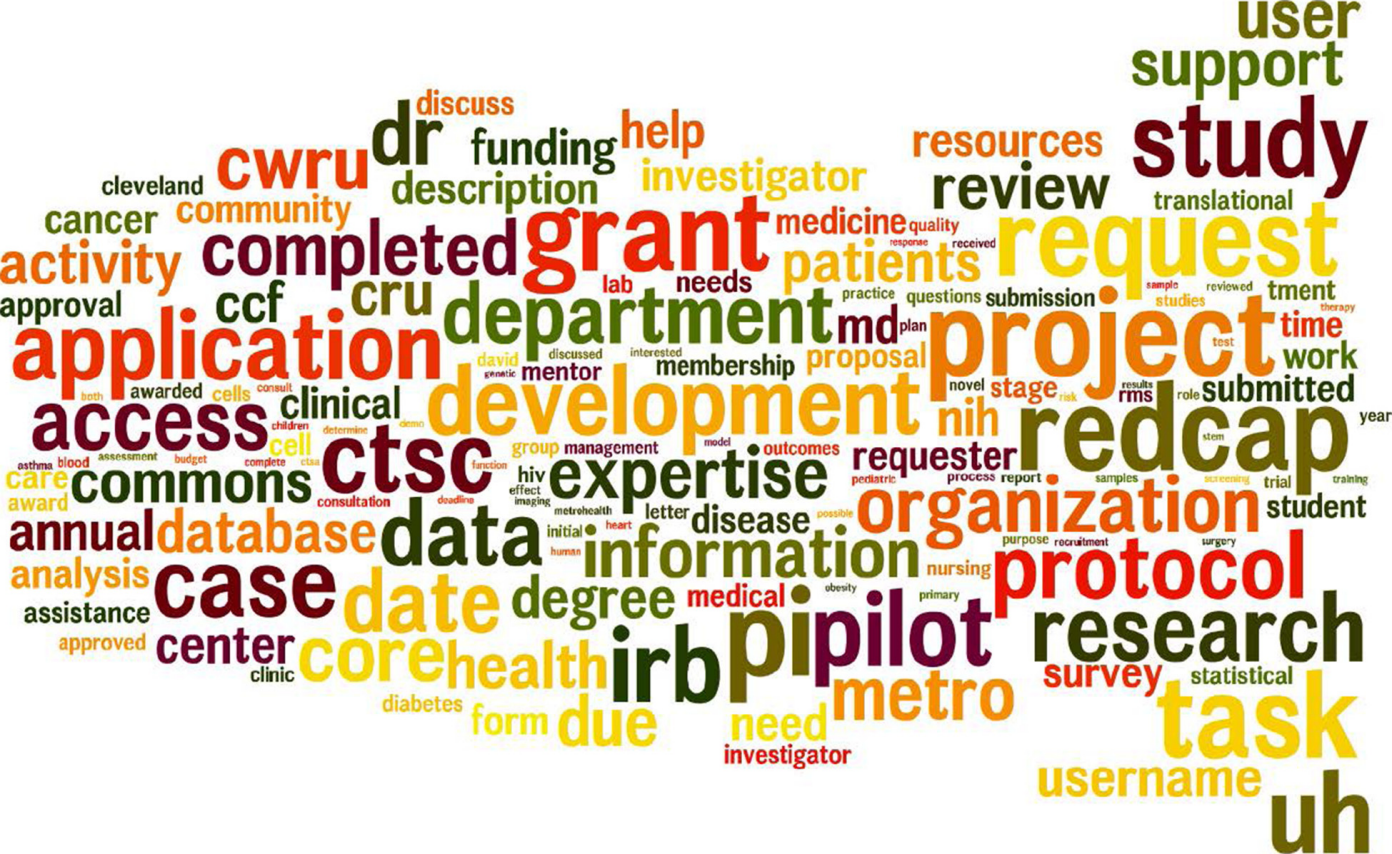
**Word cloud of project summaries and comments (top 250 words are shown; the most frequent word, “PI,” occurs 3,863 times; the least frequent word, “therapeutic,” occurs 45 times).**

RMS allows users to describe projects and discuss the progress of tasks within the system. In Figure 6, we use a word cloud to illustrate commonly discussed topics. The word cloud was generated using all of the 3,650 available projects summaries and discussions. As we expected, the primary purpose of using RMS is for collaborative research project management; hence project related words such as ‘protocol’, ‘proposal’, ‘study’, and ‘completed’ are among the most frequently used words. Investigators also often search for potential collaborators on RMS to develop new grant applications; related words are ‘grant’, ‘funding’, ‘pilot’, and ‘submission’. Investigators also use RMS to acquire CTSC resources, commonly using words such as ‘redcap’, ‘access’, ‘assistance’, and ‘resource’. Another common topic is asking support for data analysis, with words such as ‘data’, ‘statistical’, and ‘analysis’ being commonly used. Disease related topics are also common, including ‘cancer’, ‘HIV’, ‘diabetes’, and ‘asthma’. Figure 6 shows the diverse research activities supported by the RMS platform.

## Discussion

Many studies in the past two decades have discovered that a high level of research collaboration positively correlates with the quality and quantity of research outcomes [1,2,4,17,26,27,29–31]. For instance, a Swedish study focused on industrial research collaboration [32] found that compared to in-house research and publishing alone, research performed in collaboration with scientists from other institutions achieved higher impacts. A study from Germany [33] summarized three major barriers to research collaboration, including lack of management interface, incompatible work routines, and resource and budget constraints. *The lack of a management interface* is a significant barrier to researchers’ finding collaborators. Without a shared management interface, researchers often feel it is difficult to identify suitable collaborators and challenging to follow up on collaboration activities. RMS addresses this problem by providing a consistent, easy-to-access web-based interface for project management and a team of core administrators to coordinate the request process to ensure quality of service. To address the problem of *heterogeneous work routines* between different institutions, RMS provides a streamlined workflow which integrates the collaboration work routines of the affiliated institutions. Additionally, the key elements of a collaborative project, such as project goal, starting time, task due date, and expected results, are explicitly required to reduce potential ambiguity. Hence, the investigators can expect consistent consultation services supported by the system. The CTSC also provides rich expertise and administration resources to alleviate the *resource and budget constraints* of individual investigators. Domain experts on the RMS network are well aware of the responsibility of providing help to other investigators, while they also realize they can receive support from other investigators when there is a need. The mutually supported ecosystem encourages collaboration.

Although researchers have pointed out the important role of collaboration for fostering new scientific discovery, currently there are still few real systems that have been developed to support the development of research collaboration. Weng and Gennari [34] proposed a collaborative writing system to support cross-role annotation, such as that which takes place during collaborative clinical trial protocol writing. In the domain of engineering informatics, Singh et al. [35] designed the BIM framework to support 3D model based multidisciplinary collaboration. Zhang et al. [36] developed a model driven approach to support collaborative simulation for virtual project development. To address the issues of research data sharing, The National Sleep Research Resource (NSRR [37]) and the Center for SUDEP Research (CSR [38]) was recently built to provide a set of tools for researchers to conduct multisite studies and share research data. These approaches show that collaborative tools can effectively improve the specific collaborative task in the fields of clinical writing, data sharing and engineering. However, these approaches were not designed to address the challenging problem of managing diverse collaboration projects, especially coordinating experts across institution boundaries. We tackle this challenge of managing cross-institutional collaboration by creating an informatics platform that integrates expert resources and administration procedures to enable collaborative project management in a streamlined workflow. Specifically, RMS was designed to address the need for effective management of project collaboration and sharing of resources. It brings together experts from disparate fields and resources across institutions to enable users from different organizations to share services in a distributed environment.

RMS is a novel project management platform that supports and enhances collaborative clinical and translational research. Currently, most biomedical research institutions use web directories to list the expertise of researchers and describe the services of facilities. RMS improves the exiting approach by providing interactive services. The system transforms static expert profiles into collaborative procedures that allow investigators both to acquire support and to share expertise. Institutional resources, such as expert profiles, facility services, project management, and procedure administration, often scattered around different places at different divisions. RMS brings together these separate resources and creates a one-stop solution. The integrated solution bridges the gap between institutional resources, therefore facilitating research collaboration. Table 2 shows that within RMS, 176 different types of collaborative services have been created by domain experts and over 4800 tasks were finished. Diagram 4 shows that an average RMS user collaborated with 1.34 experts in 2009; while in 2012 an average user collaborated with 3.82 experts. The collaboration increased nearly 300%. Figure 5 shows that RMS supported 201 research divisions and established over 606 unique cross institutional collaborations. Compared to commercial collaboration products, such as Microsoft SharePoint and IBM Lotus Notes, RMS has several key differences and advantages. First, RMS was developed with functions that put biomedical investigators in priority. Many convenient modules were directly designed to facilitate biomedical research collaboration, such as the integrated NIH specialties, categorized facility services, embedded research outcome options, and the integrated workflow for biomedical research consultation. Second, RMS was designed with a service structure that fits to multi-institutional organizations that are common in the biomedical research field. A service can be easily shared and administrated by several partner institutions. Third, RMS is agile and cost effective. In the CTSC, only one developer is required for the technical maintenance of RMS. MS SharePoint and IBM Lotus require significantly more resources to maintain the system services [39]. Customizing these commercial products for biomedical research will require more time and development effort [40].

Another characteristic of RMS worth noting is that the system itself is a cross-disciplinary collaboration project. The RMS project team not only consists of skillful computer science developers, but also actively involves experts from the RCS, IT management, and biomedical informatics. Engaging key stakeholders from different disciplines in all steps of design and development showed itself to be an important and effective project management practice. We discussed crucial design and developed options at each stage of the process. User feedback and requested changes were captured effectively and were quickly applied to the next round of implementation. The collaborative activity involved in the development of this system helped clarify users’ needs, deepened the mutual understanding of the partners, and reduced the possibility of running into development pitfalls.

The RMS team is continuously improving the platform to provide better services and experiences for investigators and domain experts. At the early stage of implementation, some projects were set up with the assistance of the RCS, which were also considered as requests. Requests placed through RMS have been changing over time. This study focuses on analyzing the trend of RMS usage and providing descriptive analysis to the nature of collaboration in the system. We have started to develop sections of hands-on demos for investigators to help them understand more about the services provided by RMS. Recently, the CTSC BRIM has started to disseminate the RMS platform to other CTSA institutions. We have also provided consultation services and shared our experience with several institutions. Finally, the University of Rochester has started to customize and deploy a new version of RMS to serve investigators in its Medical Center.

## Conclusion

RMS is a novel system for integrating expertise and resources to support multidisciplinary and cross-institutional collaboration. In this paper, we share our experience with designing and developing the platform to support collaborative research project management and the insights we gained in the process. RMS has attracted over 1,500 users and the user base is continually growing. Our results show that the system supports rich collaboration across CTSC institutions over a very diverse range of research projects. Our empirical results indicate that RMS can foster and enhance research collaboration.

## Availability and requirements

Project name: Request Management System

Project home page: http://casemed.case.edu/ctsc/tools/rms.cfm

User instruction manual: http://casemed.case.edu/ctsc/researchers/documents/rms_manual.pdf

Quick start guide: http://casemed.case.edu/ctsc/researchers/documents/quick_start_guide.pdf

Required platform: Platform independent

Required programming language: Ruby on Rails

Academics license available: Source codes and deployment support available based on request. For details of license agreement, please contact the corresponding author: gq@case.edu
